# Cardiac Manifestations and Persistent Myocardial Dysfunction in Multisystem Inflammatory Syndrome in Children: Insights from Conventional and Strain Echocardiography

**DOI:** 10.3390/children12101383

**Published:** 2025-10-14

**Authors:** Carmen Corina Șuteu, Liliana Gozar, Nicola Șuteu, Beatrix-Julia Hack, Iolanda Muntean

**Affiliations:** 1 Department of Pediatrics III, University of Medicine, Pharmacy, Sciences and Technology “George Emil Palade” of Târgu Mureș, 540142 Târgu Mureș, Romania; carmen.suteu@umfst.ro (C.C.Ș.); liliana.gozar@umfst.ro (L.G.); iolanda.muntean@umfst.ro (I.M.); 2 Clinic of Paediatric Cardiology, Emergency Institute for Cardiovascular Diseases and Transplantation of Târgu Mureș, 540139 Târgu Mureș, Romania; 3 Faculty of General Medicine, University of Medicine, Pharmacy, Sciences and Technology “George Emil Palade” of Târgu Mureș, 540142 Târgu Mureș, Romania; nicolasuteu@yahoo.com

**Keywords:** echocardiography, speckle-tracking, global longitudinal strain, left ventricular dysfunction, SARS-CoV-2

## Abstract

**Background:** Multisystem inflammatory syndrome in children (MIS-C) is a severe post-infectious complication of SARS-CoV-2, often with cardiac involvement. Myocardial strain imaging may detect dysfunction missed by conventional echocardiography. The objectives of this study are to characterize cardiac manifestations of MIS-C and assess the value of strain imaging in children with preserved and reduced left ventricular ejection fraction (LV-EF). **Methods:** We retrospectively analyzed 22 MIS-C patients admitted between September 2020 and January 2024, all with cardiac involvement. Clinical, laboratory, and echocardiographic data—including 2D and speckle-tracking strain—were collected at the day of worst dysfunction (DWD) and discharge (DD) and compared with 22 matched controls. **Results:** Median age was 4.65 years; 59% male; 45% overweight/obese. LV systolic dysfunction (LV-EF < 50%) occurred in 54.5%, coronary abnormalities in 36.4%, and pericardial effusion in 95.5%. LV global longitudinal strain (LVGLS) was significantly lower than controls at the DWD (−15.45 ± 4.76%, *p* < 0.0001) and DD (−20.63 ± 4.66%, *p* = 0.014). Strain abnormalities persisted despite LV-EF recovery, and even patients with preserved LV-EF showed significant segmental strain reduction. LVGLS and apical infero-septal strain were strongest predictors of reduced LV-EF. **Conclusions:** MIS-C often causes systolic dysfunction and coronary changes, but strain imaging reveals persistent subclinical myocardial injury. Long-term cardiac monitoring is warranted.

## 1. Introduction

In late 2019, a novel coronavirus emerged as the cause of COVID-19, which quickly escalated into a global pandemic. The pathogen, named severe acute respiratory syndrome coronavirus 2 (SARS-CoV-2), has been linked to significant illness and death worldwide [1,2]. In children, the incidence of both symptomatic and severe infections is lower; however, the seropositive rates of SARS-CoV-2 antibody are higher compared with adults [3,4,5]. Beginning in April 2020, cases describing a novel hyperinflammatory syndrome associated with COVID-19 were first reported in the UK, Italy, and the USA [6,7,8]. These children and adolescents presented with symptoms resembling the clinical presentation of Kawasaki disease (KD), Kawasaki shock syndrome, and toxic shock syndrome induced by a cytokine storm, potentially driven by macrophage activation. The condition has been termed by the Centers for Disease Control and Prevention (CDC) and the World Health Organization (WHO) as a “multisystem inflammatory syndrome in children” (MIS-C) or “pediatric multisystem inflammatory syndrome temporally associated with SARS-CoV-2” (PIMS-TS) [9,10,11,12]. The estimated occurrence of MIS-C among children and adolescents infected with SARS-CoV-2 ranges between 0.4 and 5.5 cases per 100,000 individuals [1,13,14]. Evidence indicates that MIS-C occurs less frequently and with reduced severity in association with the Omicron variant compared with previous variants [15,16,17].

MIS-C is defined by a hyperinflammatory state accompanied by multiorgan dysfunction. It is thought to represent a delayed immune-mediated reaction, typically emerging 3 to 6 weeks after a, often asymptomatic, SARS-CoV-2 infection. Most affected children demonstrate positive serological evidence of prior infection, whereas active viral detection by RT-PCR is uncommon. This pattern supports the concept that immune dysregulation following infection, rather than the acute viral phase itself, is central to the development of MIS-C [18,19]. The precise pathways through which SARS-CoV-2 induces immune dysregulation remain under investigation. Current evidence suggests that sustained immunoglobulin G (IgG) responses may enhance monocyte activation, while persistent cytopenias—especially T cell lymphopenia—and heightened CD8+ T cell activity also appear to contribute [15]. Typical clinical features of MIS-C include prolonged fever, hemodynamic instability, and cardiac involvement. Cardiovascular complications are observed in nearly 80% of cases and may present as arrhythmias, impaired myocardial contractility, valvular regurgitation, coronary artery (CA) abnormalities, or pericardial effusion [15,20,21,22,23].

Left ventricular (LV) systolic dysfunction is the most common cardiac finding [8,17,19,23,24,25,26,27]. Recent studies reported considerably higher rates of depressed LV function (~50–60%) and CA abnormalities (~20–50%) in MIS-C patients [26,28,29,30]. Deformation parameters serve as sensitive markers for identifying subtle alterations in myocardial function; however, comprehensive assessments of cardiac mechanics using these measures remain limited in this condition. Multiple studies have documented altered strain patterns in individuals presenting with LV dysfunction [19,20,27].

The primary objective of this study is to describe a single-center experience in relation to cardiac manifestations of MIS-C and its management and early outcome in Romanian children without coronavirus disease 2019 vaccination. The second objective of the study is to analyze anatomic and functional echocardiographic manifestations of this disease in children using conventional echocardiography and 2D-strain analysis. Considering the hypothesis that a myocardial strain is a more sensitive indicator of ventricular dysfunction, we aimed to assess the global and segmental echocardiographic biventricular strain indices and to evaluate the impact of left ventricular ejection fraction (LV-EF) on the echocardiographic strain indices in MIS-C patients.

## 2. Materials and Methods

### 2.1. Study Population and Case Definition

We conducted a retrospective single-center study performed at the Pediatric Cardiology Department of the Emergency Institute for Cardiovascular Diseases and Transplantation, Târgu Mureș, a referral children’s pediatric cardiology hospital in Romania. The study included all pediatric patients who were admitted to our institution and its affiliate institutions with the diagnosis of MIS-C with associated cardiac manifestations, from September 2020 to January 2024. The diagnostic criteria of MIS-C included both the CDC and WHO case definitions [9,10]. Children with alternative causes of systemic inflammation were excluded, including those with clinical or biological evidence of sepsis. Specifically, exclusion criteria comprised a procalcitonin level of >2 ng/mL in the presence of systemic inflammatory response syndrome criteria, as well as positive blood cultures when available, in order to minimize confounding from septic cardiomyopathy. All included patients were required to have evidence of SARS-CoV-2 infection: a positive serology or positive RT-PCR. The study was approved by the local institutional review committee, and subjects gave informed consent according to the principles of the Declaration of Helsinki.

The control group consisted of healthy children among similar age groups with no structural and functional heart defects, referred to the cardiology department for the detection of an innocent systolic murmur, with echocardiographic images available in the database.

#### Clinical and Laboratory Evaluation

Patient demographics, preexisting comorbidities, clinical presentation, cardiac findings, laboratory findings, and treatment characteristics were collected on the day of worst dysfunction (DWD), defined as the day with the worst LV-EF on echocardiography. We defined myocardial injury based on 2 biomarkers, elevated N-terminal pro b-type natriuretic peptide (NT-proBNP) (>125 pg/mL) and/or positive troponin-I (>0.3 ng/mL) [31,32]. Established international reference ranges were applied to determine the cutoff values for identifying elevated laboratory markers.

### 2.2. Conventional Echocardiographic Examination

All patients underwent 2-dimensional (2D) and Doppler echocardiography using a Philips Epiq 7 ultrasound machine. Echocardiographic assessments were conducted at admission, on a daily basis throughout the acute phase, and on alternative days during the recovery phase. We defined two time points for analysis: echocardiogram on the DWD, and echocardiogram on the discharge day (DD). *Standard echocardiographic measurements* were made in accordance with the American Society of Echocardiography guidelines and included LV-EF using Simpson’s biplane method, lateral mitral annular plane systolic excursion by M-mode (MAPSE), lateral tricuspid annular plane systolic excursion by M-mode (TAPSE), peak pulsed wave tissue Doppler imaging (TDI) systolic and diastolic velocities of the lateral segment of the LV, and the LV diastolic function. MIS-C patients were divided into 2 additional groups: a normal LV-EF group (LV-EF > 50%) versus a reduced LV-EF (LV-EF < 50%). LV systolic dysfunction was defined as a LV-EF of < 50% and further based on the patient`s lowest LV-EF as moderately reduced LV-EF (LV-EF 40–49%), reduced LV-EF (LV-EF 30–39%), and severely reduced LV-EF (LV-EF < 30%).

**Figure 1:**
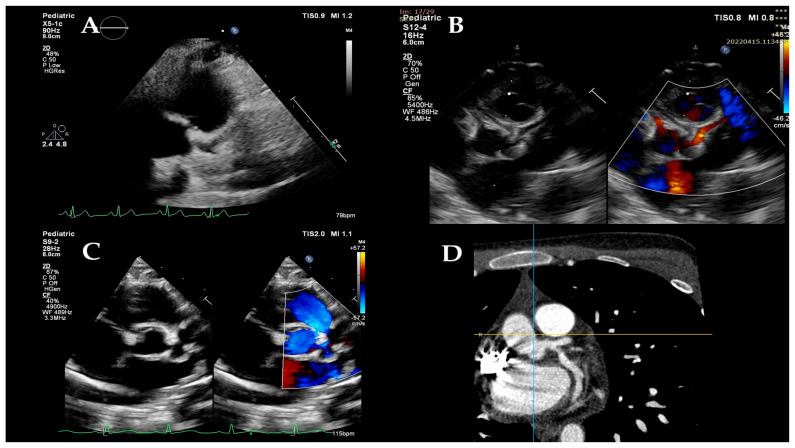
**Coronary arteries.** Two-dimensional echocardiography confirming the dilation of the left coronary artery (**A**) and color Doppler flow through the left coronary artery (**B**). Two-dimensional echocardiography showing left coronary artery ectasia (**C**). CT angiography image of the dilated left coronary artery—red arrow (**D**).

Coronary artery dilation was identified when the z-score of the affected segment exceeded 2 using the Boston z-score system; then, we classified the CA anomalies in accordance with the American Heart Association guidelines (Figure 1) [33].

### 2.3. Strain Imaging

**Figure 2:**
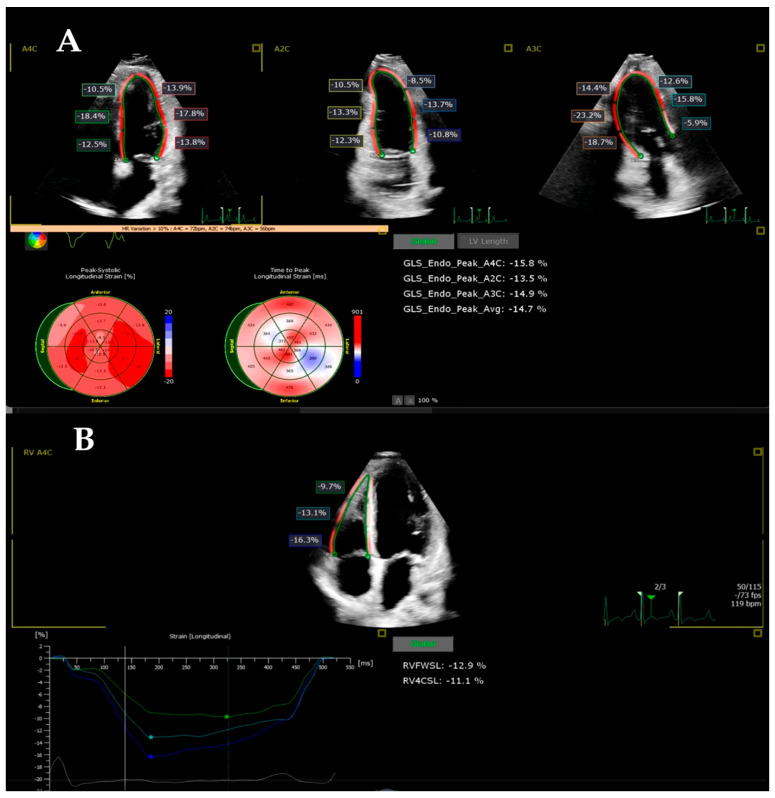
Quantification of left and right ventricular longitudinal strain image. (**A**) Left ventricular global longitudinal strain (LVGLS) analysis from apical 4-, 2-, and 3-chamber views. Strain values are diffusely reduced (average LVGLS-14.7%), indicating impaired systolic function. The bull’s-eye plot highlights segmental reduction, predominantly in the infero-septal and anterior segments. (**B**) Right ventricular free-wall longitudinal strain (RVFWLS) and 4-chamber strain analysis demonstrating decreased RV systolic function (RVFWLS-12.9%, RV4CSL-11.1%).

Myocardial deformation was assessed by biventricular speckle-tracking analysis (LV AutoStrain and right ventricle (RV) AutoStrain) using the four-chamber acquisitions, recorded with a frequency (frame rate) of over 60 Hz and optimal quality. Acquisitions obtained at the time of the clinical echocardiogram were analyzed offline with the strain software using Philips QLAB 15 software, by a single individual with over 3 years of experience. The method has been described in a previously published study [34]. The echocardiography images of the patients included in the study were analyzed, measuring the LV global longitudinal strain (LVGLS), the RV free-wall longitudinal strain (RVFWLS), the RV four-chamber longitudinal strain (RV4CLS), and the biventricular segmental strain. The interventricular septum and the walls were separated into three segments: basal infero-septal (BIS), medium infero-septal (MIS), apical infero-septal (AIS), basal antero-lateral (BAL), medium antero-lateral (MAL), apical antero-lateral (AAL), basal RV (BRV), medium RV (MRV), and apical RV (ARV) (Figure 2). The values were compared with the echocardiographic parameters measured in the control group.

### 2.4. Statistical Analysis

Statistical analyses were conducted using SPSS software, version 20 (IBM SPSS Statistics 20). The data were categorized as either nominal or quantitative variables. Nominal variables were expressed as numbers or percentages. Quantitative variables were expressed by mean ± standard deviation or by median and percentiles (25; 75%), whenever suitable. The Shapiro–Wilkins test was used with the purpose of testing for normality of distribution of quantitative variables. Comparisons of means or medians between two groups were assessed using the *t*-test or the Mann–Whitney test, as appropriate. For analyses involving three groups, a general linear model with Bonferroni post hoc correction was applied. A *p*-value of <0.05 was regarded as statistically significant.

## 3. Results

Over the period from September 2020 to January 2024, a total of 22 MIS-C patients were included in the study. The cohort was mainly male (*n* = 13; 59.09%), with a median age of 4.65 years (range 2.37–15.25 years). Table 1 shows demographic data for our study groups: MIS-C patients on the DWD and control group. Compared with age-marched controls, MIS-C patients had lower SaO2 (*p* = 0.037), increased heart HR (*p* = 0.0001), and impaired LV-EF (*p* = 0.035) (Table 1).

### 3.1. Description of the Children from the MIS-C Group on the DWD

Clinical, laboratory, and therapeutic characteristics of the children from the MIS-C group on the DWD are summarized in Table 2. Most patients were previously healthy, and 10% of them had overweight/obesity. All children presented with persistent fever (>38.5 °C) (median of 5 days of fever); other common signs and symptoms included digestive symptoms (95.45%), respiratory symptoms (50%), arthralgia (40.90%), myalgia (27.27%), and limping (9.09%). Clinical signs suggestive of Kawasaki disease, rash (54.54%), lymphadenopathy (59.09%), edema (45.45%), conjunctivitis (40.90%), and stomatitis (36.36%), were frequent. The median duration of symptoms at the time of presentation was 3 days (range 2–5 days). All children presented cardiac manifestations: LV dysfunction (54.54%), mitral valvular dysfunction (90.90%), pericardial effusion (95.45%), and CA involvement (36.36%). Shock was present in one patient. The EKG was not specific, with sinus tachycardia (95.45%), diffuse T wave inversions (100%), and ST segment elevation (54.54%). Worst cardiac dysfunction (as determined by echo LV-EF) occurred on a median of 6 days (range 4–7 days) after the onset of illness. Four patients of the cohort required admission to intensive care during their hospital stay, and there were no deaths. In 19 patients, RT-PCR was negative, but IgG antibodies to COVID-19 were positive.

All patients presented in a hyperinflammatory state characterized by elevated C-reactive protein (median, 113.81 mg/L ± 84.22), serum ferritin (median, 389.25 ng/mL; IQR, 185.00–593.00), interleukin-6 (median, 15.39 pg/mL; IQR, 11.50–65.60), and lymphopenia (median, 1570.00 cells/μL; IQR, 1050.00–2440.00). Cardiac biomarkers were elevated; the peak of biomarker abnormality was on the DWD. The parameters checked were NT-proBNP (median, 953.00 pg/mL; IQR, 425.00–8776.00), and high-sensitivity troponin I (hsTnI) (median, 84.00 ng/L; IQR, 16.00–385.00). Additional characteristic findings included the hypercoagulation state, with elevated D-dimers (median, 394.70 μg/mL; IQR, 330.19- 464.90).

Medications used included intravenous immunoglobulin (IVIG) (100%), corticosteroids (81.81%), aspirin (81.81%), anticoagulants (77.27%), inotropic support (13.63%), angiotensin-converting enzyme inhibitor—ACEi (63.63%), beta-blockers (13.63%), and diuretics. Most MIS-C patients exhibited rapid clinical improvement. Median patient discharge occurred after 15 days (range 11–21 days) and clinically corresponded to normalization of the inflammatory markers and improving the LV-EF.

Conventional echocardiographic parameters for all MIS-C patients on the DWD are shown in Table 3. More than half of the MIS-C patients (54.54%) showed impaired LV-EF on the DWD. The lowest LV-EF was moderately reduced (LV-EF: 40–50%) in seven patients (58.33%) and severely reduced (LV-EF < 30%) in three patients (13.63%). Regional wall motion abnormalities with basal septal dyskinesia were described in all MIS-C patients. LV diastolic function was assessed by E/A ratio and tissue Doppler imaging. Mitral regurgitation was present in 20 patients (90.90%) and was more than mild on the DWD in 6 patients (27.27%). Moderate/severe tricuspid regurgitation was present in two patients on the DWD. Pericardial effusion was detected in 21 patients (95.45%).

On the DWD, echocardiographic evaluation showed dilation (z-score > 2) in any CA in eight patients (36.36%). CA ectasia (z-score: 2 ≤ z < 2.5) was noted in one patient and involved the left main coronary artery (LMCA). Mild segmental CA aneurysms (z-score: 2.5 ≤ z < 5) were detected in six patients (27.27%); the most affected arteries were the LMCA (five patients), left anterior descending (LAD) (five patients), and right coronary artery (RCA) (two patients). One patient developed giant CA aneurysms (z-score ≥ 10), along the left circumflex artery (LCx) (z-score: 11.76) and LAD (z-score: 29.51). During hospitalization, most CA abnormalities completely returned to normal, persisting at discharge only in the case of the patient who had developed giant aneurysms.

### 3.2. Follow-Up of the Echocardiographic Strain Indices in the MIS-C Patients (DWD Versus DD)

**Figure 3:**
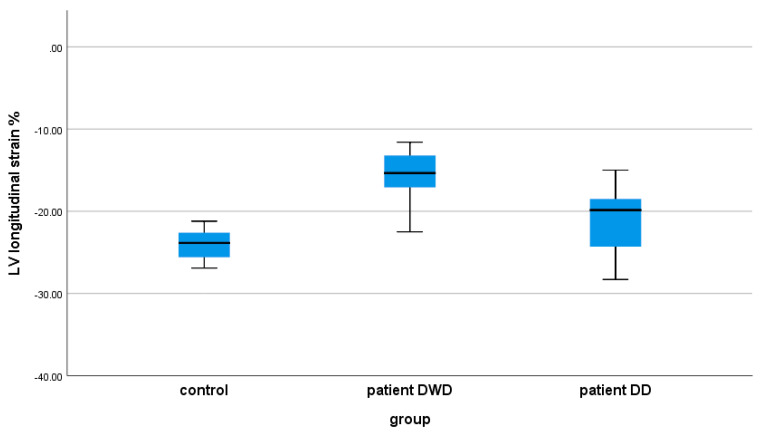
Left ventricular longitudinal strain in controls and MIS-C patients on the day of worst dysfunction (DWD) and on the day of discharge (DD).

**Figure 4:**
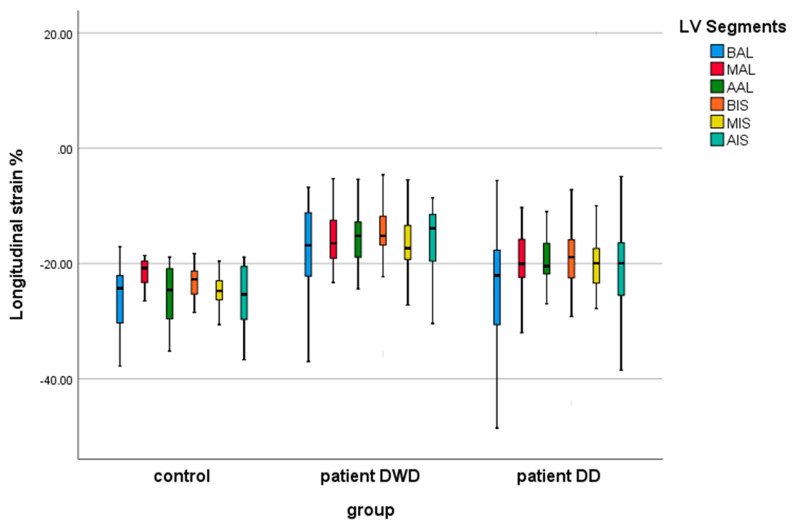
Left ventricular segmental strain indices in controls and MIS-C patients on the day of worst dysfunction (DWD) and on the day of discharge (DD).

**Figure 5:**
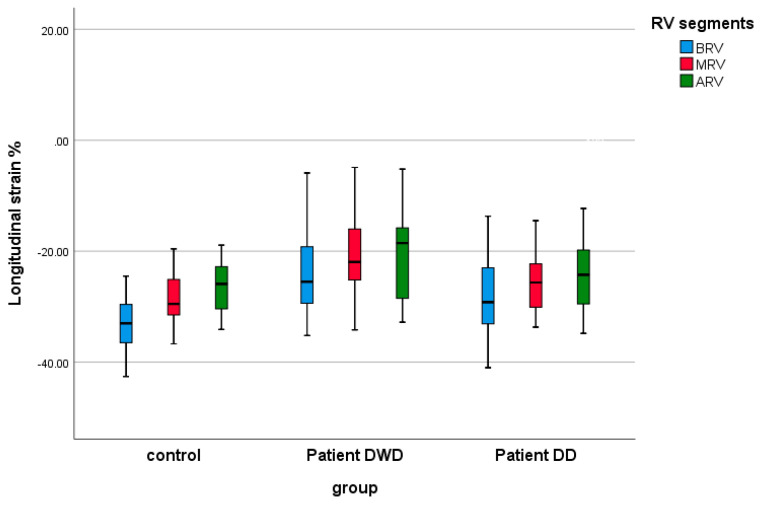
Right ventricular segmental strain indices in controls and MIS-C patients on the day of worst dysfunction (DWD) and on the day of discharge (DD).

Table 4 shows the results of the echocardiographic global and segmental strain indices in controls and the MIS-C patients on the DWD and on the DD. Comparing the age-matched control group with MIS-C patients, we found evidence of a significant lower peak LVGLS, both on the DWD (*p*: 0.0001) and on the DD (*p*: 0.014) (Figure 3). In the MIS-C group, the biventricular segmental strain analysis demonstrated significantly reduced strain indices in all LV and RV segments on the DWD compared with the control group (Figure 4 and Figure 5). On the DD, LV segmental strain indices remained significantly reduced in MIS (*p*: 0.004), AIS (*p*: 0.024), and AAL (*p*: 0.001) (Figure 4), and the RV segmental LS was still impaired on BRV (*p*: 0.032) (Figure 5).

When comparing the deformation parameters in the MIS-C group on the DWD vs. DD, we demonstrated worse LV function on the DWD by significantly lower LVGLS (*p*: 0.0001) (Table 4, Figure 4). The LV segments most affected by the myocardial injury were AIS (*p*: 0.015), BAL (*p*: 0.028), and AAL (*p*: 0.025) (Table 4, Figure 5). RV global and segmental strain indices showed no significant correlation in the MIS-C group on the DWD vs. DD.

### 3.3. The Impact of LV EF on the Echocardiographic Strain Indices

On the 22 MIS-C patients, 12 (54.54%) demonstrated reduced LV systolic function (LV-EF < 50%). Compared with the normal group, both the normal LV-EF group (*p*: 0.0001) and the reduced LV-EF group (*p*: 0.0001) had significantly lower LVGLS (Table 5). There was no significant difference in the RV global strain indices (RVFWLS, RV4CLS) between the three groups. It was especially noticeable that all LV and RV segments showed significant LS reduction in the group with reduced LV-EF compared with the control group (Table 5). In the normal LV-EF group, myocardial injury was demonstrated by abnormal segmental strain indices in the infero-septal segments (BIS (*p*: 0.027), MIS (*p*: 0.0001), AIS (*p*: 0.002), medium and apical antero-lateral segments (MAL (*p*: 0.014), AAL (*p*: 0.0001)) as well as in BRV (*p*: 0.006).

Comparing the echocardiographic global and segmental strain indices between MIS-C patients with normal LV-EF and those with reduced LV-EF, we found that the strongest predictors associated with systolic LV disfunction were LVGLS (*p*: 0.009) and AIS LS (*p*: 0.047) (Table 5).

During the admission period, serial echocardiography assessments demonstrated significant improvement in LV-EF; only one patient had a moderately depressed LV-EV on the DD. However, when comparing MIS-C patients with initially reduced LV-EF to controls on the DD, we founded evidence of impaired global and segmental strain indices: LVGLS (*p*: 0.0001), BISLS (*p*: 0.008), AISLS (*p*: 0.007), MALLS (*p*: 0.005), AALLS (*p*: 0.001), BRVLS (*p*: 0.026) (Table 6). Furthermore, compared with MIS-C patients with normal LV-EF, the reduced LV-EF group in MIS-C had a considerably lower LVGLS (*p*: 0.003), BISLS (*p*: 0.029), and MALLS (*p*: 0.015) on the DD. There was not a significant difference in the RV global and segmental strain indices between the MIS-C groups on the DD (Table 6).

## 4. Discussion

MIS-C is considered an uncommon yet serious complication of COVID-19 in the pediatric population. While it can develop across all age groups, the majority of cases are reported in previously healthy children aged 6 to 12 years, usually appearing a few weeks after SARS-CoV-2 exposure [1,14]. Comorbid conditions were reported in 27–36% of patients; the most communal comorbidities were obesity and asthma [1,8,17]. There is increasing recognition of the frequency and severity of cardiac involvement, which plays a major role in both acute management and prognosis. The etiology of cardiovascular involvement in MIS-C is multifactorial, including cardiac myocytes and vascular endothelium injury attributed to an intense and dysregulated inflammatory response related to high-level cytokine release (cytokine storm), viral invasion of cardiac tissue, hypoxia, and ischemia caused by CA involvement [15,35]. Studies by Feldestein LR et al., Kobayashi R et al., Valverde I et al., and Dhanalakshmi K et al. had reported cardiovascular involvement in 80%, 68%, 68%, and 63% of patients, respectively [8,20,21,36].

The current study described detailed clinical and echocardiographic findings in 22 MIS-C children with associated cardiac manifestations. Our MIS-C patients share similar features with other reported studies including previously healthy children manifesting fever, inflammation, and multisystem involvement [6,7,23]. The clinical presentation was common, with a predominance of gastrointestinal and mucocutaneous symptoms, but we noted a smaller age range in the MIS-C cohort (median age of 4.56 years) compared with previous reported data [19,20,23,24]. Many patients in our cohort (n = 10, 45.45%) were overweight or obese. Similar results were also observed by Riphagen S et al., Dufort EM et al., Capone CA et al. [6,17,23]. The majority of cases (n = 19, 86.36%) had positive immunoglobulin G serology, suggesting a post-infectious, immune-mediated physiopathology. All patients in our study presented in a hyperinflammatory state, with elevated proinflammatory cytokines such as IL-6 and elevated NT-proBNP and hsTnI levels, biomarkers that have been used to define myocardial injury. In addition, endothelial injury and activation of the coagulation cascade have been documented by elevated levels of D-dimer. The cardiovascular manifestations included systolic ventricular disfunction, CA abnormalities, mitral regurgitation, pericardial effusion, and cardiac electric abnormalities consisting of sinus tachycardia (95.45%), diffuse T wave inversions (100%), and ST segment elevation (54.54%). Studies by Israel V et al. and Jashvanth HJ et al. showed abnormal ST segment and T wave changes in 28.5% and 22%, respectively [21,37].

Echocardiography was fundamental to diagnosis and decision making. In this study, we observed that the principal observation in the acute phase of MIS-C was myocardial injury presenting with a myocarditis-like pattern, which could be mild or subclinical, especially in patients with preserved LV-EF. Our study showed greater evidence of LV systolic dysfunction, with over half of MIS-C patients (54.54%) having a reduced LV-EF, typically worsening by day 6 of illness. On the DWD, the echocardiography showed moderately depressed LV-EF (LV-EF: 40–49%) in most cases (n = 7; 58.33%); only three children (13.63%) presented severely reduced LV-EF (LV-EF < 30%). LV systolic dysfunction has been described in a large percentage of children diagnosed with MIS-C [20,21,30,38,39]. Results comparable to ours were observed by Theocharis P et al. in a study that included a similar number of children as our cohort. They reported initially reduced LV-EF (LV-EF < 55%) in half of the patients with serial echocardiograms demonstrating the worst LV dysfunction on day 7 of illness [24]. The study performed by Feldstein LR et al., which assessed the LV dysfunction in a large cohort included 503 MIS-C patients, reported initial mildly depressed LV-EF (defined as LV-EF: 45–54%) in a similar percentage (55.2%) observed in our study [30]. Higher rates of impaired LV-EF (~50–60%) have also been reported in a case series that included only patients with severe forms of MIS-C [26,28,29]. Studies by Matsubara D et al., Grimaud at al., and Jashvanth HJ et al. reported a higher incidence of severe cases with cardiogenic shock [19,22,37]. In contrast to prior studies that showed a higher incidence of shock, our study demonstrated a less severe clinical spectrum despite the greater evidence of systolic LV dysfunction. In our cohort, the cardiogenic shock was present in one patient, and four patients were admitted to the intensive care unit.

Deformation parameter analysis may allow recognition of ventricular dysfunction, particularly in patients with subclinical myocardial injuries. Previous studies in cardiac MRI-proven viral myocarditis have shown that strain indices can independently predict ventricular dysfunction in patients with preserved LV-EF [38]. Several studies have reported abnormal strain patterns in MIS-C patients with LV dysfunction [19,20]. In our single-center study, we demonstrated that both LVGLS and all biventricular segmental strain indices were significantly decreased on the DWD in MIS-C patients compared with the age-matched control group. We also identified LVGLS and AISLS as the strongest predictors associated with impaired LV-EF. Furthermore, even in normal LV-EF group, global (LVGLS) and segmental (BISLS, MISLS, AISLS, MALLS, AALLS, BRVLS) strain assessments showed evidence of impaired LV systolic function compared with healthy controls. The combination of these strain abnormalities and elevated cardiac biomarkers is strongly suggestive of underlying myocarditis. This finding is consistent with results of prior studies by Matsubara D et al. and Kobayashi R et al., wherein peak strain and strain rates have been noted to be abnormal despite normal LV-EF [19,20]. Similar results were also observed by Theocharis P et al. in a small study involving 20 patients with MIS-C who underwent both echocardiography and cardiac MRI. They reported (3D) LV-EF of < 55% in half of the patients, but almost all patients displayed abnormal strain indices at the time of admission [24].

In our population, the treatment protocol, including immunomodulatory therapy (IVIG) and corticosteroids, was linked with clinical improvement, reduction in inflammatory markers and rapid improvement in LV systolic function. Previous case series have reported recovery of LV-EF within a range of days to a few weeks of diagnosis suggesting that MIS-C ventricular dysfunction likely results from severe inflammation more often than from ischemia or direct virus-mediated myocardial damage [19,20,22,23,26,27,30]. The current study demonstrated the normalization of LV-EF after a median length of hospitalization of 15 days. However, on the DD, we observed significant persistent global (LVGLS) and segmental strain (AISLS, BALLS, AALLS) abnormalities. Similarly to our results, studies by Kobayashi R et al. and Theocharis P et al. identified ongoing ventricular dysfunction with abnormal strain patterns in MIS-C patients persisting beyond the acute phase [20,24]. These findings suggest that subclinical LV dysfunction persists in some patients despite normalization of inflammatory markers and underline the importance of serial strain monitoring in identifying patients with ongoing myocardial disease.

Clinical Implications of LV Strain in Pediatric Practice: Our findings support growing evidence that STE provides clinically meaningful information on myocardial function in children with MIS-C, even when conventional echocardiographic indices such as LV-EF are preserved. Given its ability to detect subclinical myocardial dysfunction, incorporation of LVGLS into routine pediatric echocardiographic evaluation—particularly in high-risk populations such as MIS-C—may enhance early recognition of myocardial involvement and guide management decisions. However, widespread adoption in clinical practice should take into account potential limitations, including availability of the software, dependence on optimal image quality, variability across vendors, and the impact of arrhythmias or irregular heart rates on strain measurements. These factors underscore the importance of standardized acquisition protocols and careful interpretation before STE can be recommended as a universal component of routine pediatric echocardiography.

Coronary artery involvement has been described in MIS-C; however, the pathophysiology has not been elucidated [8,17,26]. The CA dilation in MIS-C could reflect a physiological response to amplified myocardial oxygen demand caused by a highly proinflammatory milieu or may be attributable to destruction of the arterial wall by inflammatory cells as is seen in KD [19,30,35,40,41]. The frequency of CA abnormalities varies significantly among reports, but comparing the results is often difficult due to the inconsistent criteria used for defining coronary dilatation and aneurysms in children with MIS-C. In Theocharis P’s study, defining CA dilation by the presence of a z-score of > 2 in the affected segment, in accordance with reference standard published by Dallaire F, found a higher incidence (60%) of patients with enlarged CA [24]. Belhadjer Z used a CA z-score adjusted for body temperature and reported 17% of patients with CA dilation; no coronary aneurysm has been observed [27]. Verdoni L detected left CA aneurysms in two (20%) patients using the echocardiographic criteria for coronary aneurysm of > 4 mm (in patients ≥ 5 years of age) [7]. The CA involvement in MIS-C patients was quite infrequent (4%) in a study reported by Matsubara D et al. To evaluate CA, they used the Boston z-score system and classified CA abnormalities based on the AHA statement paper [19]. Future studies based on standardized protocols for defining coronary artery dilatation and aneurysms using z-scores will lead to a much more accurate interpretation of the results. In contrast to the study performed by Matsubara, but using the same criteria for reporting coronary dimensions, we found a higher incidence of CA abnormalities (36.36%). We noticed the predominance of mild segmental CA aneurysms (z-score: 2.5 ≤ z < 5) (27.27%); the most affected arteries were LMCA, LAD, and RCA. In our cohort, only one patient had segmental giant CA aneurysms (z-score ≥ 10) involving LCx and LAD. We applied current treatment protocols for MIS-C, the regimen included IVIG, corticosteroids, antiplatelet, and anticoagulation therapy. This treatment protocol was associated with favorable clinical outcomes, with a complete return to normal CA in almost all patients. This is in line with previously published data that have noted the mild severity and quicker resolution of CA aneurysms in MIS-C than in typical KD [19,20,24].

**Limitations:** This study has several limitations that warrant consideration. First, it was conducted at a single tertiary center, involved a relatively small cohort, and had a retrospective design, which may limit the generalizability of the findings. The small sample size may also have reduced the statistical power to detect subtle differences between subgroups. Second, all strain analyses were performed offline by a single experienced operator to minimize variability, and therefore inter- and intra-observer reproducibility could not be assessed. Third, follow-up was limited to the in-hospital period, and thus, the persistence of subclinical myocardial dysfunction beyond discharge could not be systematically evaluated. Finally, STE, although highly promising, may be influenced by technical factors such as image quality, frame rate, and cardiac rhythm disturbances, which could affect strain reproducibility. Future multicentre, prospective studies with larger cohorts and standardized protocols, including reproducibility analysis and long-term follow-up, are needed to validate our findings and clarify their prognostic significance.

## 5. Conclusions

MIS-C is a new multisystemic inflammatory syndrome in relation to COVID-19 infection in children. We found that ventricular systolic dysfunction and CA abnormalities are more common in the acute phase of MIS-C. Subtle abnormalities in myocardial deformation were observed even in patients with preserved LV-EF, indicating the presence of subclinical myocardial injury. Although the immediate outcome is favorable, longitudinal cardiac follow-up is essential to fully understand the evolution and prognosis of this novel disease, as the physiopathology of cardiovascular involvement is not fully understood and there is echocardiographic evidence of ongoing myocardial dysfunction.

## Figures and Tables

**Table 1:** General characteristics of the children from the study groups (control group and MIS-C patients on day of worst dysfunction). The bold are statistically significant.

Parameter	Control n= 22	Patient DWD n = 22	*p*
Age (year)	5 (3.00, 15.00)	4.65 (2.37, 15.25)	0.762
Gender (male%)	16 (72.72%)	13 (59.09%)	
Weight (kg)	18.5 (13.62, 55.75)	21 (12.3, 63.00)	0.763
Height (cm)	103.5 (87.25, 158.75)	118.04 ± 43.05	0.763
BSA (m^2^)	0.74 (0.59, 1.58)	0.79 (0.56, 1.75)	1
BSAz	0.04 ± 0.66	0.29 ± 0.69	0.223
BMI (kg/m^2^)	17.88 ± 3.02	18.89 ± 4.75	0.403
BMIz	0.10 ± 1.07	0.10 ± 1.90	0.993
SaO2 (%)	99 (99.00, 100.00)	98 (96.75, 99.00)	0.037
sBP (mmHg)	101.00 ± 13.35	102.54 ± 14.57	0.716
sBPz	0.08 ± 0.55	0.24 ± 0.90	0.479
dBP (mmHg)	53.00 ± 10.04	57.13 ± 10.32	0.185
dBPz	−0.03 ± 0.65	0.42 (−0.74, 1.26)	0.132
HR (b/min)	88.54 ± 15.54	115.40 ± 23.37	**0.0001**
HRz	−0.05 ± 0.76	1.88 ± 1.27	**0.0001**
LV-EF (%)	58 (53.90, 67.50)	48.02 ± 12.04	**0.035**

Data are presented as means ± SD or median (25th, 75th percentiles) or as numbers (percentages; BMI, body mass index; BMIz, body mass index z-score; BP, blood pressure; BSA, body surface area; BSAz, body surface area z-score; dBP, diastolic blood pressure; dBPz, diastolic blood pressure z-score; HR, heart rate; HRz, heart rate z-score; LV-EF, left ventricular ejection fraction; SaO2: oxygen saturation; sBP, systolic blood pressure; sBPz, systolic blood pressure z-score.

**Table 2:** Clinical, laboratory and therapeutic characteristics of the children from the MIS-C group on day of worst dysfunction (DWD).

Parameters	*n*/Mean/Median	%
**General**		
overweight/obesity	10	45.45
other comorbidities	2	9.09
total day fever	5 (4.00, 6.00)	
day presentation	3.00 (2.00, 5.00)	
DWD	6.00 (4.00, 7.00)	
ICU admission	4	18.18
DD	15.00 (11.00, 21.00)	
**Organ system involvement**		
hypotension/sock	1	4.54
cardiac	22	100.00
renal	3	13.63
respiratory	11	50.00
hematologic	19	86.36
gastrointestinal	21	95.45
dermatologic	12	54.54
neurologic	2	9.09
total systems	1	4.54
**Symptoms**		
fever	22	100.00
conjunctivitis	9	40.90
stomatitis	8	36.36
rash	12	54.54
edema	10	45.45
lymphadenopathy	13	59.09
digestive symptoms	21	95.45
respiratory symptoms	11	50.00
arthralgia	9	40.90
myalgia	6	27.27
limping	2	9.09
fatigue	19	86.36
shock	1	4.54
**Cardiac manifestations**		
LV dysfunction (LV-EF: <50/49–40/<39)	12 (n = 7/n = 2/n = 3)	54.54 (31.81/9.09/13.63)
mitral regurgitation	20	90.90
pericardial effusion	21	95.45
coronary involvement	8	36.36
arrhythmias	1	4.54
EKG changes	22	100.00
sinus tachycardia	21	95.45
diffuse T wave inversions	22	100.00
ST segment elevation	12	54.54
COVID testing		
RT PCR	3	13.63
serology	19	86.36
**Laboratories**		
hsTnI (ng/L)	84.00 (16.00, 385.00)	
NT-proBNP (pg/mL)	953.00 (425.00, 8776.00)	
leukocytes count (cells/μL)	9144.44 ± 4485.88	
lymphocyte count (cells/μL)	1570.00 (1050.00, 2440.00)	
neutrophil count (cells/μL)	6487.22 ± 3965.78	
erythrocyte count (cells/μL)	4.32 ± 0.70	
platelet count (cells/μL)	250,977.77 ± 151,885.12	
hemoglobin (g/dL)	11.45 (10.40, 14.10)	
CRP (mg/L)	113.81 ± 84.22	
interleukin-6 (pg/mL)	15.39 (11.50, 65.60)	
ferritin (ng/mL)	389.25 (185.00, 593.00)	
fibrinogen (mg/dL)	394.70 (330.19, 464.90)	
D-dimer (μg/mL)	1785.00 (867.00, 2560.00)	
albumin (g/dL)	3.57 ± 0.81	
creatinine (mg/dL)	0.59 ± 0.27	
ALT (Ui/mL)	36.75 (20.00, 44.00)	
LDH (Ui/mL)	279.50 (220.00, 365.00)	
**Treatment**		
Immunoglobulin infusion	22	100.00
Intravenous steroids	18	81.81
Anticoagulation Enoxaparin	17	77.27
Aspirin	17 + 1 clopidogrel	81.81
Inotropic support	3	13.63
Interleukin receptor antagonist	0	0.00
ACEi Lisinopril	13 + 1 captopril	63.63
Spironolactone	20	90.90
Hydrochlorothiazide	6	27.27
Furosemide	3	13.63
Beta-blockers	3	13.63

Data are presented as means ± SD or median (25th, 75th percentiles) or as numbers (percentages); ACEi, angiotensin-converting enzyme inhibitor; ALT, alanine transaminase; CRP, C-reactive protein; DD, day of discharge; DWD, day of worst dysfunction; EKG, electrocardiogram; hsTnI, high-sensitivity troponin I; ICU, intensive care unit; LDH, lactate dehydrogenase: LV, left ventricle; NT-proBNP, N-terminal pro b-type natriuretic peptide; RT-PCR, reverse transcription polymerase chain reaction.

**Table 3:** Conventional echocardiographic characteristics of the children from the MIS-C group on day of worst dysfunction (DWD).

Parameters	*n*/Mean/Median	%
LVEF	48.02 ± 12.04	
LVEF < 50%	12	54.54
MAPSE (mm)	11.12 ± 4.09	
TAPSE (mm)	15.35 ± 5.17	
Mi E/A ratio	1.45 ± 0.49	
Mi E/e`lat ratio	0.06 (0.06, 0.08)	
Mi s`lat	7.57 ± 1.94	
Mi s`lat (z-score)	−0.61 ± 0.97	
Mi e`lat	13.15 ± 4.00	
Mi e`lat (z-score)	−0.75 (−1.77, −0.02)	
Mi a`lat	7.04 ± 2.38	
Mi a`lat (z-score)	0.73 ± 1.54	
basal septal dyskinesia	22	100.00
mitral valve regurgitation	20	90.90
mitral valve regurgitation > mild	6	27.27
tricuspid valve regurgitation	17	77.27
tricuspid valve regurgitation > mild	2	9.09
pericardial effusion	21	95.45
coronary artery (CA) dilation	8	36.36
LMCA (z-score)	1.54 ± 1.52	
LAD (z-score)	1.20 (0.90 ± 3.60)	
LCx (z-score)	0.88 (0.80 ± 1.30)	
proximal RCA (z-score)	1.03 (0.57 ± 1.40)	
CA z-score z < 2	14	63.63
CA z-score 2 ≤ z < 2.5	1	4.54
CA z-score 2.5 ≤ z < 5	6	27.27
CA z-score 5 ≤ z < 10	0	0.00
CA z-score z ≥ 10	1	4.54

CA, coronary artery; LAD, left anterior descendent; LCx, left circumflex; LV-EF, left ventricular ejection fraction; LMCA, left main coronary artery; MAPSE, mitral annular plane systolic excursion; Mi, mitral; RCA, right coronary artery; TAPSE, tricuspid annular plane systolic excursion.

**Table 4:** Echocardiographic global and segmental strain indices in controls and MIS-C patients on the day of worst dysfunction (DWD) and on the day of discharge (DD). The bold are statistically significant.

Parameter	Control n= 22	Patient DWD n = 22	Patient DD n = 22	c vs. pDWD	c vs. pDD	pDWD vs. pDD
LV GLS (%)	−24.23 ± 2.23	−15.45 ± 4.76	−20.63 ± 4.66	**0.0001**	**0.014**	**0.0001**
LV Max D (mm)	62.09 ± 17.64	63.63 ± 19.15	64.04 ± 18.35	1.000	1.000	1.000
LV Max S (mm)	49.00 ± 14.01	53.86 ± 17.58	53.13 ± 16.75	0.969	1.000	1.000
RVFWLS (%)	−27.13 ± 13.37	−21.94 ± 7.86	−25.90 ± 6.76	0.248	1.000	0.549
RV4CLS (%)	−23.90 ± 10.77	−18.30 ± 9.90	−21.34 ± 10.87	0.248	1.000	1.000
BIS LS (%)	−23.51 ± 4.02	−15.54 ± 6.39	−19.89 ± 7.88	**0.0001**	0.183	0.077
MIS LS (%)	−25.45 ± 4.10	−16.70 ± 5.10	−18.51 ± 9.82	**0.0001**	**0.004**	1.00
AIS LS (%)	−25.62 ± 5.33	−15.38 ± 5.48	−20.66 ± 7.02	**0.0001**	**0.024**	**0.015**
BAL LS (%)	−26.12 ± 5.56	−18.19 ± 9.01	−25.14 ± 10.45	**0.010**	1.00	**0.028**
MAL LS (%)	−22.13 ± 3.85	−15.45 ± 5.08	−18.83 ± 6.05	**0.0001**	0.151	0.136
AAL LS (%)	−25.20 ± 4.86	−15.26 ± 4.90	−19.30 ± 4.99	**0.0001**	**0.001**	**0.025**
BRV LS (%)	−33.55 ± 5.32	−23.85 ± 7.91	−27.72 ± 8.43	**0.0001**	**0.032**	0.259
MRV LS (%)	−28.68 ± 4.55	−21.11 ± 7.55	−24.99 ± 6.83	**0.001**	0.187	0.15
ARV LS (%)	−26.24 ± 4.55	−18.59 ± 10.26	−23.64 ± 7.50	**0.005**	0.819	0.107

AAL, apical antero-lateral; AIS, apical infero-septal; ARV, apical right ventricular; BAL, basal antero-lateral; BIS, basal infero-septal; BRV, basal right ventricular; LV GLS, left ventricular global longitudinal strain, LV Max D, maximum left ventricular diameter in diastole; LV max S, maximum left ventricular diameter in systole; MAL, medium antero-lateral; MIS, medium infero-septal; MRV, medium right ventricular; RVFWLS, right ventricular free-wall longitudinal strain; RV4CLS, right ventricular four-chamber longitudinal strain.

**Table 5:** Comparison of echocardiographic global and segmental strain indices between three groups on the day of worst dysfunction (DWD): controls, MIS-C patients with normal LV EF, and those with reduced LV EF. The bold are statistically significant.

Parameter	Control n = 22	Patient nEF n = 10	Patient rEF n = 12	*p* (c vs. nEF)	*p* (c vs. rEF)	*p* (nEF vs. rEF)
LV GLS (%)	−24.23 ± 2.23	−17.94 ± 4.68	−13.39 ± 3.90	**0.0001**	**0.0001**	**0.009**
LV Max D (mm)	62.09 ± 17.64	65.00 ± 17.74	62.50 ± 17.64	1.000	1.000	1.000
LV Max S (mm)	49.00 ± 14.01	53.70 ± 14.59	54.00 ± 20.39	1.000	1.000	1.000
RVFWLS (%)	−27.13 ± 13.37	−24.46 ± 6.81	−19.84 ± 8.33	1.000	0.214	0.995
RV4CLS (%)	−23.90 ± 10.77	−21.69 ± 4.99	−15.49 ± 12.16	1.000	0.081	0.494
BIS LS (%)	−23.51 ± 4.02	−18.16 ± 7.58	−13.36 ± 4.41	**0.027**	**0.0001**	0.103
MIS LS (%)	−25.45 ± 4.10	−17.72 ± 4.83	−15.85 ± 5.38	**0.0001**	**0.0001**	1.000
AIS LS (%)	−25.62 ± 5.33	−18.39 ± 6.33	−12.88 ± 3.09	**0.002**	**0.0001**	**0.047**
BAL LS (%)	−26.12 ± 5.56	−21.21 ± 11.10	−15.68 ± 6.25	0.256	**0.001**	0.255
MAL LS (%)	−22.13 ± 3.85	−17.07 ± 4.14	−14.10 ± 5.56	**0.014**	**0.0001**	0.380
AAL LS (%)	−25.20 ± 4.86	−16.72 ± 4.95	−14.05 ± 4.73	**0.0001**	**0.0001**	0.622
BRV LS (%)	−33.55 ± 5.32	−25.10 ± 7.43	−22.80 ± 8.46	**0.006**	**0.0001**	1.000
MRV LS (%)	−28.68 ± 4.55	−23.20 ± 6.69	−19.37 ± 8.06	0.074	**0.0001**	0.463
ARV LS (%)	−26.24 ± 4.55	−22.94 ± 6.55	−14.97 ± 11.59	0.762	**0.0001**	0.052

AAL, apical antero-lateral; AIS, apical infero-septal; ARV, apical right ventricular; BAL, basal antero-lateral; BIS, basal infero-septal; BRV, basal right ventricular; LV GLS, left ventricular global longitudinal strain, LV Max D, maximum left ventricular diameter in diastole; LV max S, maximum left ventricular diameter in systole; MAL, medium antero-lateral; MIS, medium infero-septal; MRV, medium right ventricular; RVFWLS, right ventricular free-wall longitudinal strain; RV4CLS, right ventricular four-chamber longitudinal strain.

**Table 6:** Comparison of echocardiographic global and segmental strain indices between three groups on the day of discharge (DD): controls, MIS-C patients with normal LV EF and those with reduced LV EF. The bold are statistically significant.

Parameter	Control n = 22	Patient nEF n = 10	Patient rEF n = 12	*p* (c vs. nEF)	*p* (c vs. rEF)	*p* (nEF vs. rEF)
LV GLS (%)	−24.23 ± 2.23	−23.34 ± 2.84	−18.37 ± 4.76	1.000	**0.0001**	**0.003**
LV Max D (mm)	62.09 ± 17.64	65.90 ± 17.28	62.50 ± 19.82	1.000	1.000	1.000
LV Max S (mm)	49.00 ± 14.01	53.20 ± 15.20	53.08 ± 18.62	1.000	1.000	1.000
RVFWLS (%)	−27.13 ± 13.37	−28.09 ± 6.13	−24.09 ± 6.97	1.000	1.000	1.000
RV4CLS (%)	−23.90 ± 10.77	−25.87 ± 3.40	−17.57 ± 13.50	1.000	0.304	0.219
BIS LS (%)	−23.51 ± 4.02	−23.59 ± 8.72	−16.80 ± 5.79	1.000	**0.008**	**0.029**
MIS LS (%)	−25.45 ± 4.10	−17.80 ± 13.54	−19.11 ± 5.77	**0.035**	0.075	1.000
AIS LS (%)	−25.62 ± 5.33	−23.25 ± 7.85	−18.51 ± 5.72	0.932	**0.007**	0.228
BAL LS (%)	−26.12 ± 5.56	−27.96 ± 10.52	−22.80 ± 10.23	1.000	0.807	0.457
MAL LS (%)	−22.13 ± 3.85	−22.44 ± 5.41	−15.82 ± 6.98	1.000	**0.005**	**0.015**
AAL LS (%)	−25.20 ± 4.86	−20.76 ± 5.22	−18.09 ± 4.67	0.066	**0.001**	0.631
BRV LS (%)	−33.55 ± 5.32	−29.09 ± 8.48	−26.56 ± 8.59	0.317	**0.026**	1.000
MRV LS (%)	−28.68 ± 4.55	−26.19 ± 4.32	−24.00 ± 8.46	0.806	0.092	1.000
ARV LS (%)	−26.24 ± 4.55	−26.33 ± 5.15	−21.40 ± 8.59	1.000	0.092	0.190

AAL, apical antero-lateral; AIS, apical infero-septal; ARV, apical right ventricular; BAL, basal antero-lateral; BIS, basal infero-septal; BRV, basal right ventricular; LV GLS, left ventricular global longitudinal strain, LV Max D, maximum left ventricular diameter in diastole; LV max S, maximum left ventricular diameter in systole; MAL, medium antero-lateral; MIS, medium infero-septal; MRV, medium right ventricular; RVFWLS, right ventricular free-wall longitudinal strain; RV4CLS, right ventricular four-chamber longitudinal strain.

## Data Availability

The data presented in this study are available on request from the corresponding author.

## Abbreviations

The following abbreviations are used in this manuscript:

KD	Kawasaki disease
CDC	Centers for Disease Control and Prevention
WHO	World Health Organization
MIS-C	Multisystem inflammatory syndrome in children
PIMS-TS	Pediatric multisystem inflammatory syndrome temporally associated with SARS-CoV-2
RT-PCR	Reverse-transcriptase polymerase chain reaction
IgG	Immunoglobulin G
CA	Coronary artery
LV	Left ventricular
BMI	Body mass index
BP	Blood pressure
BSA	Body surface area
D	Diastole
HR	Heart rate
LV-EF	Left ventricular ejection fraction
S	Systole
Y	Year
Z	z-score
DWD	Day of worst dysfunction
NT-proBNP	N-terminal pro b-type natriuretic peptide
2D	2-dimensional
DD	Echocardiogram on the discharge day
MAPSE	Lateral mitral annular plane systolic excursion by M-mode
TAPSE	Lateral tricuspid annular plane systolic excursion by M-mode
TDI	Peak pulsed wave tissue Doppler imaging
RV	Right ventricle
LVGLS	LV global longitudinal strain
RVFWLS	RV free-wall longitudinal strain
RV4CLS	RV four-chamber longitudinal strain
BIS	Basal infero-septal
MIS	Medium infero-septal
AIS	Apical infero-septal
BAL	Basal antero-lateral
MAL	Medium antero-lateral
AAL	Apical antero-lateral
BRV	Basal RV
MRV	Medium RV
ARV	Apical RV
hsTnI	High-sensitivity troponin I
IVIG	Intravenous immunoglobulin
ACEi	Angiotensin-converting enzyme inhibitor
ALT	Alanine transaminase
CRP	C-reactive protein
DD	Day of discharge
EKG	Electrocardiogram
ICU	Intensive care unit
LDH	Lactate dehydrogenase
LMCA	Left main coronary artery
LAD	Left anterior descending
RCA	Right coronary artery
LCx	Left circumflex artery
Mi	Mitral
LVGLS	Left ventricle global longitudinal strain
ARV	Apical right ventricular
BRV	Basal right ventricular
LV Max D	Maximum left ventricular diameter in diastole
LV max S	Maximum left ventricular diameter in systole
MRV	Medium right ventricular
RVFWLS	Right ventricular free-wall longitudinal strain
RV4CLS	Right ventricular four-chamber longitudinal strain
